# Stroke rehabilitation: A call to action in Saudi Arabia

**DOI:** 10.17712/nsj.2016.4.20160075

**Published:** 2016-10

**Authors:** Saad M. Bindawas, Vishal S. Vennu

**Affiliations:** *From the Department of Rehabilitation Sciences, College of Applied Medical Sciences, King Saud University, Riyadh, Kingdom of Saudi Arabia*

## Abstract

Stroke is a major cause of death and other complications worldwide. In Saudi Arabia, stroke has become an emerging health issue leading to disability and death. However, stroke care including rehabilitation services, in Saudi Arabia lags behind developed countries. Stroke rehabilitation is an essential recovery option after stroke and should start as early as possible to avoid potential complications. The growing evidence on stroke rehabilitation effectiveness in different health care settings and outcome measures used widely are reviewed in this call to action paper.

The Kingdom of Saudi Arabia (KSA) is the largest nation in the Arabian Peninsula, stretching over a zone of 2,150,000 square kilometers and boasting a population of more than 28 million.^1^ Stroke is a quickly developing issue, and an imperative reason for disease and death in Saudi Arabia. It has been well documented that stroke is a major cause of death and functional impairment worldwide.^2^ Thus, stroke stands out among the most basic social and financial medical issues in the Kingdom.^1^ The type of stroke in Saudi Arabia is comparable from that reported in western countries, with an inconsistency in the low recurrence of subarachnoid discharge (SAH).^1^ The most important risk factors for stroke in Saudi Arabia are similar to findings in other studies, such as hypertension, diabetes mellitus, heart disease, and smoking.^1^ From the epidemiological perspective, the first reported stroke incidence rate was 29.8 among 100000 Saudi citizen annually.^1^ Saudi Arabia territorial reports demonstrate that stroke is one of the main reasons for morbidity and mortality in the Gizan region, an extensive provincial territory as which has lower stroke occurrence than the reported rates in urban areas.^3^ For health care services for patients with stroke in Saudi Arabia, a recent study found that stroke care on the national level falls behind developed countries.^4^ For example, this study found that out of 350 hospitals, only 2 have a specialized stroke team. The stroke rehabilitation program remains an essential element of recovery after stroke, and should be started as early as possible to avoid potential complications and death. Thus, in this call to action paper, we sought to review the recent literature on stroke rehabilitation effectiveness in different health care settings and the outcome measures that are commonly used in these settings by using the International Classification of Functioning, Disability and Health (ICF) classification of health and health-related domains.^5^

## Stroke rehabilitation

As indicated by the ICF model developed by the World Health Organization (WHO) in 2001, stroke rehabilitation can be described as a health procedure that “aims to facilitate people with health state experiencing or likely to experience disability to attain optimal functioning in interaction with the environment”.^5^ Generally, it is an umbrella for several services to help patients with stroke in improving their physical, psychosocial, and vocational potential, with consideration of the physiologic and environmental limitations. However, various advances have recently been made in the prevention, treatment, and rehabilitation of stroke worldwide. These advances have elicited a significant decrease in the population-based death rate over the past decade.^6^ Generally, stroke rehabilitation programs remain the essential recommended treatment option for post-stroke functional limitations and disability.^7^

## The components of stroke rehabilitation

**Figure 1:**
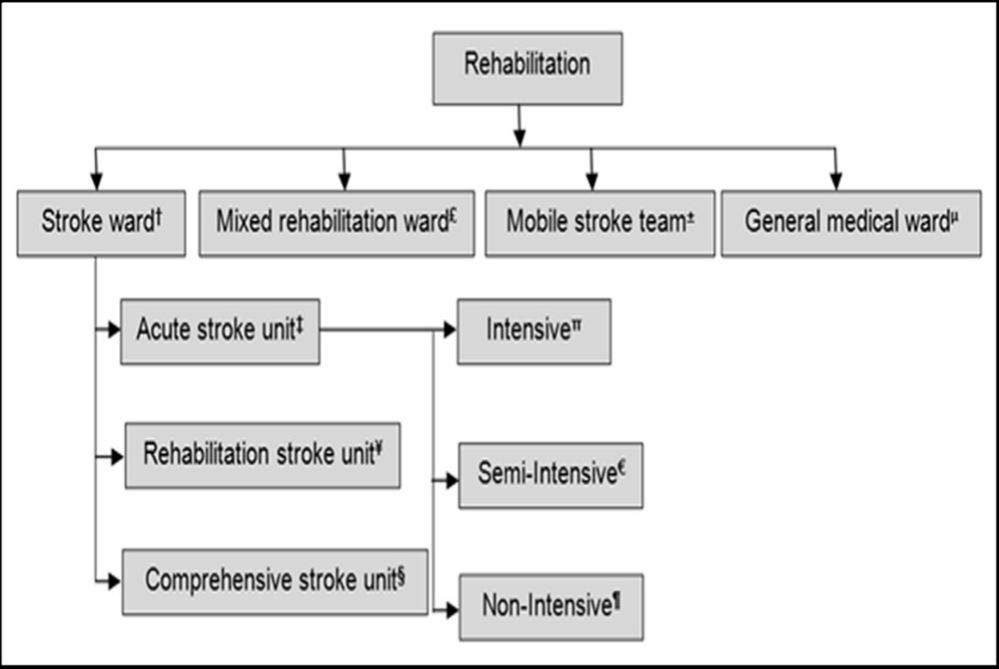
Elements of a stroke unit associated with improved outcome. ^†^A multidisciplinary team including a specialist nursing staff. ^‡^Stroke patients are assessed in the acute care hospital by a physical therapist or rehabilitation physician who decides the level of mobility impairment and the capacity to endure treatment. Patients are discharged early within 7 days. ^π^Model of consideration with constant checking, high medical attendant staff, and life support. ^€^Model of consideration with constant checking, high medical attendant staff, yet no life support facilities. ^¶^Model with no high medical attendant staff or life support facilities. ^¥^A wide scope of therapeutic and rehabilitative services and settings that give consideration to patients admitted after intense administration in a hospital setting. Patients are accepted after a period of usually of 5 to 7 days or more. ^§^Rehabilitation offered for at least several weeks if necessary. ^£^A multidisciplinary group gives a non-specific rehabilitation service, not only watching over stroke patients. ^±^A multidisciplinary group (barring nursing staff) gives care in an assortment of settings. ^µ^An intense medicinal or neurology ward without routine multidisciplinary information.

The primary goals of stroke rehabilitation are to regain independence and improve quality of life by minimizing the limitations of activities of daily living (ADL).^8^ Structured stroke care should consider the early timing of rehabilitation, a qualified rehabilitation team, and duration of rehabilitation, which are important elements that have been distinguished as advancing better general outcomes for patients with stroke.^9^ Evidence from systematic reviews support that organized stroke rehabilitation units, and more prominent intensities of rehabilitation are associated with enhanced improved functional outcomes compared with mixed rehabilitation units, general units, and mobile stroke units.^10^ This suggests that neurological rehabilitation alone does not represent the level of useful changes observed in stroke rehabilitation.^9^ Rehabilitation services regardless of the setting are found to be associated with better functional outcomes.^10^ Examples for these settings where rehabilitation services are provided are stroke wards, ambulatory settings, and others, as shown in **Figure 1**. There is strong evidence supporting the beneficial effects of early admission to stroke rehabilitation units within 24-48 hours after stroke, to enhance functional outcomes.^6^ However, screening for potential admission to stroke rehabilitation units should be performed when the patient’s health state is stable.^11^ The clinician performing the assessment should be specialized in stroke rehabilitation or have extensive experience in neurorehabilitation; as the patient’s type and condition severity and classification are vital determinants of disabilities and functional abilities, along with capacity to learn and physical action continuance.^11^ Stroke rehabilitation services consist of a team of professionals’ that help with the patient’s physical needs, and focus on cognitive, emotional, and vocational skills.^12^ Yet, evidence has shown that stroke rehabilitation professionals cooperating in groups are more effective in enhancing functional recovery and quality of life compared with a single specialty (intra discipline).^13^ The stroke professional team members, and their responsibilities are summarized in **Table 1**. In terms of the duration, there is no agreement on the ideal time needed to complete the rehabilitation program for patients with stroke. Stroke rehabilitation programs change extensively between settings and units. According to the American Congress of Rehabilitation Medicine, duration is defined as “period of time during which a solitary session is directed”.^14^,^15^ Also, the duration of each stroke rehabilitation session varies depending on several factors such as the recovery, severity, related complications, and responsiveness to therapy.^15^ Although some stroke survivors recover quickly, most need some form of stroke rehabilitation for a longer time, conceivably months or years.

**Table 1:** Stroke rehabilitation team members and their responsibilities.

Member	Responsibility
Patient and family	Characterizes objectives, assumes control over own rehabilitation program and long-term disability administration
Rehabilitation nurse	Creates a restorative environment, case administration, family instruction, skin and bowel/bladder care
Rehabilitation social worker	Appraisal and administration of family and community assets, discharge arrangements, case administration
Physician	Therapeutic administration of inability, oversees comorbid conditions; included less outside of serious rehabilitation settings
Occupational therapist	Appraisal and treatment of self-consideration aptitudes; upper extremity disability, splints, and assistive devices
Physical therapist	Appraisal and treatment of mobility issues; quality, adaptability, balance, continuance, coordination, help with mobility
Orthotics/prosthetics	Suggests, plans, manufactures, and gains individualized equipment
Speech and language pathologist	Appraisal and administration of communication disorders, swallowing
Psychologist	Appraisal and administration of cognitive, behavioral, and effective status; connects with the perceptual-motor and language status
Dietetics and nutrition	Appraisal and administration of the dietary state, extraordinary eating regimens, enteral and parental feeding
Recreation therapist	Appraisal and administration of leisure preferences, adoptions, and integration into the therapeutic plan
Optometrist	Appraisal and administration of low vision weaknesses and disability

## The effectiveness of stroke rehabilitation

In this section, we aim to briefly summarize recent studies on the effectiveness of stroke rehabilitation programs in different settings. Effective stroke rehabilitation programs are defined by an interdisciplinary specialist team, working cohesively and closely providing an extensive rehabilitation program to every patient.^16^ These programs alter regarding the types of therapy offered in addition to their intensity and length of time. In general, successful stroke rehabilitation relies on: physical elements (including the severity of stroke in terms of both cognitive and physical impacts); emotional causes (such as motivation and mood, and ability to stick with rehabilitation activities outside of therapy sessions); social aspects (for example, the support of family); and curative factors (such as an early start to rehabilitation and the skills of the stroke rehabilitation team).^12^ Results from most of the recently published randomized controlled trials (RCTs) provide strong evidence that stroke rehabilitation at the acute stroke stage,^17^ sub-acute stroke stage,^18^ and chronic stroke stage^19^ decreased death and disability. In a recent RCT,^9^ stroke care combined with comprehensive rehabilitation and early supported discharge was associated with enhanced survival rate and functional outcome, and a reduced requirement for institutionalization, and length of hospital stay.^9^ The Stroke Unit Trialists Collaboration’s (SUTC) systematic review of RCTs^20^ demonstrated that stroke patients who obtained treatment in a stroke rehabilitation unit are more likely to be alive and have a lower risk of being dependent compared with patients who obtained care in general wards. The results of this review also showed that stroke survivors may continue to benefit from intervention after the sub-acute phase.^20^ A recent systematic review that included 96 studies with 10,401 participants investigated the effects of different physical rehabilitation approaches.^21^ More than half of these studies were carried out in China. Of 96 included studies, 27 studies were on stroke rehabilitation, and all of them provided evidence on the effectiveness of stroke rehabilitation in improving functional recovery. Furthermore, 12 studies found stroke rehabilitation more effective in improving motor function compared with usual care.

## Stroke rehabilitation in the outpatient setting

Outpatient stroke rehabilitation is defined as a type of treatment where patients go to a clinic or hospital to attend sessions and then return home the same day.^22^ However, for some economical and sociodemographic factors, along with an expanding number of stroke survivors, there is growing enthusiasm for outpatient stroke rehabilitation.^23^ Outpatient facilities can be components of a larger hospital facility and provide access to healthcare services including stroke rehabilitation programs. Patients regularly spend a few hours, frequently 3 days weekly at the facility in treatment sessions and return home afterward.^23^ A recent Canadian trial^24^ was conducted to investigate the role of a combination of outpatient and inpatient rehabilitation programs on cardiovascular fitness and walking ability. All 50 participating stroke patients received 60-minute physical therapy sessions 5 times weekly as inpatients for 6 weeks, and 3 times weekly as outpatients for another 6 weeks. They found that those patients who received the stroke rehab program including the body-weight-supported treadmill training had better outcomes compared with the usual stroke care. In a smaller pilot randomized trial, researchers investigated the effects of outpatient rehabilitation on walking and balance for patients with chronic stroke.^11^ Rehabilitation was provided for 4 weeks in a tertiary neurological hospital in China. The results suggested that short outpatient rehabilitation programs were viable in enhancing balance and walking function after stroke.

## Stroke rehabilitation in the inpatient setting

An inpatient stroke rehabilitation facility is defined as a multidisciplinary team that exclusively oversees stroke patients in a ward at least one week after stroke.^20^ These facilities may be independent or constitute some portion of a larger hospital system. Patients stay in these facilities, generally for 2 to 3 weeks, and participate in comprehensive rehabilitation programs. Normally, these programs incorporate no less than 3 hours of element treatment per day, for 5 or 6 days a week. A previously published RCT conducted in Germany evaluated the effects of post-acute inpatient rehabilitation after stroke.^25^ All 30 participating stroke patients received treatment over a period of 2 weeks. Results from this trial indicated that inpatient stroke rehabilitation has positive effects on mobility. Furthermore, a pilot RCT from Australia aimed at testing motor and cognitive functions in patients with acute post-stroke after a cerebral infarction.^26^ All 44 participating stroke patients were randomly assigned to 5, one-hour sessions for one week of therapist-supervised practicing of daily tasks. The results of this study showed a major improvement in motor function. A more recent systematic review, was conducted to investigate which variables can predict functional independence at discharge from inpatient rehabilitation facilities.^27^ This review included 27 studies reporting Barthel Index (BI) or Functional Independence Measure (FIM) scores. The results summarized that functional ability improved in patients after post-stroke inpatient rehabilitation, and that this can be predicted by variables such as age and history of stroke.

## Stroke rehabilitation in a nursing facility

A nursing facility (NF) is defined as a facility where a client is admitted, and services are provided by a multidisciplinary professional team, aimed at restoring function.^28^ These facilities differ technically, yet they provide some kind of rehabilitation services. If the service is provided, it is for fewer hours than the hospital setting as outlined above (approximately 4 hours per patient over 3-5 working days) in most NFs.^28^ Patients with stroke after the acute phase are referred either to independent rehabilitation centers or NFs based on sociodemographic and economic factors such as age, general conditions, and level of impairment.^28^ A small, but well-designed RCT from a single NF in St. Louis in the United States of America (USA), included 26 older patients.^29^ Patients were randomized to enhanced medical rehabilitation or standard-of-care rehabilitation. Along with functional outcomes, therapy intensity, and engagement were measured. The results from this trial found that NF patients in the intervention group had better functional outcomes, higher intensity, and patient engagement compared with the standard-of-care rehabilitation. A recent systematic review,^30^ included 19 articles, and examined the different predictive variables associated with discharge destination of acute stroke patients after hospitalization, including NFs.^30^ This review revealed that functional dependency or comorbidity was the main factor that influenced admission in a NF after acute care. The effect of other variables such as age, gender, and race differed between studies, and remains under debate.

## Stroke rehabilitation in the home-based setting

Home-based stroke rehabilitation is characterized as a complex package of care provided by a clinician or nurse aiming at either avoiding the need for admission to hospital, or empowering timely and more virtual discharge and follow-up at home.^31^ Home-based rehabilitation takes into account extraordinary adaptability, such that patients can tailor their program of rehabilitation around their individual needs and schedules. This care is often most appropriate for individuals who require treatment by a standard rehabilitation professional.^31^ A previous trial on 71 therapy practices was conducted in northern Germany over 2 years to compare the effects of rehabilitation programs in homes or standard-of-care for patients with upper limb dysfunction after stroke.^32^ This trial included 156 patients, 85 of them were assigned to receive a rehabilitation program in their homes, and 71 patients were assigned standard therapy. This study revealed that home-based therapy was more effective than conventional therapy in improving quality of movement and motor function. A systematic review of 11 RCTs investigated the functional benefits of a home-based rehabilitation program for community-dwelling people with stroke.^33^ The results of this review demonstrated that home-based rehabilitation was very effective at 6 weeks and 3 months, but less clear at 6 months. Also, individual studies of this review reported increased care satisfaction in favor of home-based rehabilitation.

## The role of telerehabilitation and technology for patients with stroke

Telerehabilitation is defined as the delivery of rehabilitation services including clinical assessment and clinical therapy over telecom systems, and the web.^34^ These are an alternative way of delivering stroke rehabilitation services using information and communication technologies between the healthcare professional and the patient in a remote area. These communications may occur through a variety of technologies such as the telephone, internet-based videoconferencing, and sensors such as pedometers. Virtual reality (VR) is one example for using technology for rehabilitation purposes, where the patient completes treatment inside a computer-produced virtual environment, and information is transmitted to the specialist.^35^ Virtual reality is defined as an interaction that allows users to interact with a multisensory stimulated environment and receive real time feedback on performance between advanced computer-technology and the users with a computer-generated environment in a naturalistic fashion.^36^ The use of VR has risen as a new treatment approach in stroke rehabilitation settings over the last 10 years.^37^ This approach might be advantageous as it provides an opportunity to practice activities that cannot be practiced inside the confines of a clinical environment. Furthermore, there are a few components of VR that may imply that patients invest more energy in treatment: for example, the activity might be more motivating.^37^ A study conducted in Spain^38^ was carried out to evaluate the clinical effectiveness of using VR as telerehabilitation program in improving balance after stroke. All 30 participating stroke patients received 20, 45-minute training sessions with the telerehabilitation program, conducted 3 times a week in the clinic or at home. The results revealed that VR based telerehabilitation programs promote the loco-motor skills associated with balance in the same way as in-clinic interventions. Similarly, a systematic review of 11 RCTs^39^ on the effects of telerehabilitation on improving functions for patients with stroke in their homes, found limited to moderate evidence that telerehabilitation of all approaches has equal effects with conventional rehabilitation in improving ADL and motor functions, In another recent systematic review,^37^ RCTs were included to compare VR effects on upper limb functions and impact on gait, cognitive function, and ADL after stroke; with an alternative intervention or no intervention. The review’s results found that VR was more effective than conventional therapy in improving upper limb function based on 12 studies, and significantly more effective than no therapy in improving upper limb function based on 9 studies. The use of VR also improved ADL function when compared to more conventional therapy approaches based on 8 studies. Furthermore, a systematic review with meta-analysis^40^ including 15 trials was published recently to compare the effects of VR based rehabilitation verses standard rehabilitation, or VR based rehabilitation added to the standard rehabilitation regimen.^40^ Some beneficial effects of VR based rehabilitation in walking speed, balance, and mobility outcomes in stroke survivors compared with standard rehabilitation were identified. The results also showed greater benefit in mobility when VR based rehabilitation was added to standard rehabilitation. On the other hand, there are many other technologies that can be used with stroke rehabilitation programs, such as using robotics. Robot-assisted rehabilitation is a developing technology field that aims to be utilized in the stroke rehabilitation setting.^41^ Robotic technology represents a highly repetitive and task-oriented feasible tool to administer in stroke rehabilitation. The potential of robotic technology in stroke rehabilitation might offer extensive advantages, not just regarding cost, but also for the researchers with an approach inspired from evidence-based practice. Evidence from a systematic review^42^ that included 11 RCTs found that robot-assisted therapy had similar effects to conventional therapy on upper limb motor recovery, strength, motor control, and ADL.^42^

## Stroke rehabilitation outcome measures using the International Classification of Functioning, Disability, and Health model

**Figure 2:**
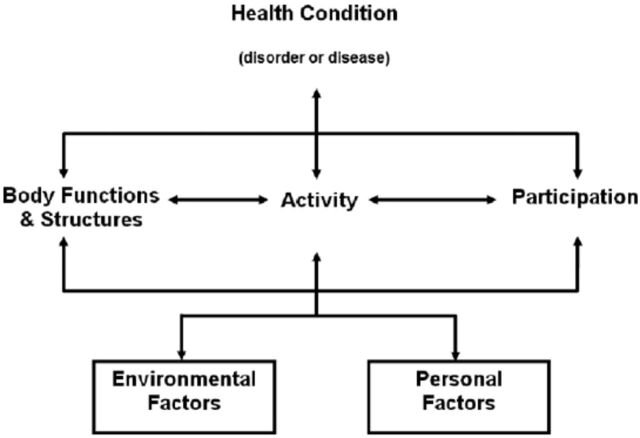
Interactions between segments of the International Classification of Functioning, Disability, and Health model.

There are many outcome measures to be used in stroke rehabilitation.^43^ In 2001, the WHO developed a framework for measuring health and disability at both the individual and population levels, called the International Classification of Functioning, Disability and Health, known more commonly as ICF (**Figure 2**).^5^ The ICF is a brief, important, and accurate instrument that can be used in stroke rehabilitation to provide a multi-dimensional approach to describing stroke patients functioning and disability, and to help to organize this information.^44^ Body functions can be defined as physiological functions of body systems, while activities and participation can be defined as execution of a task or action by an individual and involvement in a life situation.^45^ In **Table 2**, we summarized all possible examples of measures that may be used in stroke rehabilitation in any settings, according to the 3 ICF domains. All of these measures were then described with some details such as objectives, advantages, and disadvantages in **Appendix 1**.

**Table 2:** A selection of outcome measures that have demonstrated construct validity in stroke rehabilitation by using the International Classification of Functioning, Disability, and Health domains.^44^,^45^

Body structure and function	Activities	Participation
1. Beck Depression Inventory	1. Action Research Arm Test	1. Canadian Occupational Performance Measure
2. Behavioral Inattention Test	2. Barthel Index	2. EuroQoL Quality of Life Scale
3. Canadian Neurological Scale	3. Berg Balance Scale	3. Assessment of Life Habits (LIFE-H)
4. Clock Drawing Test	4. Box and Block Test	4. London Handicap Scale
5. Frenchay Aphasia Screening Test	5. Chedoke McMaster Stroke Assessment Scale	5. Medical Outcomes Study Short-Form 36
6. Fugl-Meyer Assessment	6. Chedoke Arm and Hand Activity Inventory	6. Nottingham Health Profile
7. General Health Questionnaire-28	7. Clinical Outcome Variables Scale	7. Reintegration to Normal Living Index
8. Geriatric Depression Scale	8. Functional Independence Measure	8. Stroke Adapted Sickness Impact Profile
9. Hospital Anxiety and Depression Scale	9. Frenchay Activities Index	9. Stroke Impact Scale
10. Line Bisection Test	10. Motor Assessment Scale	10. Stroke Specific Quality of Life
11. Mini-Mental State Examination	11. Nine-hole Peg Test	
12. Modified Ashworth Scale	12. Rankin Handicap Scale	
13. Montreal Cognitive Assessment	13. Rivermead Mobility Scale	
14. Motor-free Visual Perception Test	14. Rivermead Motor Assessment	
15. National Institutes of Health Stroke Scale	15. Six-Minute Walk Test	
16. Orpington Prognostic Scale	16. Timed Up and Go	
17. Stroke Rehabilitation Assessment of Movement	17. Wolf Motor Function Test	
	18. Dynamic Gait Index	

## General recommendations to improve stroke rehabilitation programs in Saudi Arabia

From this review, the optimal stroke rehabilitation parameters (frequency, type, duration, intensity) could not be established. It will continue to be a major healthcare provocation in Saudi Arabia. However, many components of the stroke burden can be prevented and managed, including implementing the following recommendations for rehabilitation professionals, policy makers, and for future research:

## 1. Recommendations for rehabilitation professionals

Rehabilitation professionals such as physical/occupational therapists and others in Saudi Arabia are not necessarily well trained in managing patients with stroke. Therefore, we believe there is a need for strengthening education in stroke rehabilitation in the curricula for all health care professionals, including education on the specific topics related to stroke prevention, management, and rehabilitation. To be able to offer high quality services for patients with stroke, post-professional programs should be introduced and supervised by the Saudi Commission for Health Specialties. We believe it is time for professionals such as physical therapists in Saudi Arabia to establish residency and fellowship programs, especially those that work with patients with stroke such as neurology and geriatrics.^46^ One of the challenges for rehabilitation professionals is using evidence-based practice. Continuing education is an important factor in providing high quality services for patients with stroke and other conditions.^47^ Therefore, it is important for all members of the rehab team involved in managing patients with stroke to be trained in evidence-based practice though continuing education activities.^48^

## 2. Recommendations for policy makers

Policy makers can play a major role toward translating science into practice in the field of stroke care. For better health care services for patients with stroke in Saudi Arabia, we believe there is a need for a national framework to help guide the utilization and generation of evidence to support decision making for complex, multifactorial public health challenges, including stroke prevention, management, and rehabilitation. This national framework will close the evidence-to-practice gap on the national level, by increasing the dissemination of evidence-based stroke rehabilitation findings between the rehabilitation professionals, other health care professionals, policymakers, and the public in general. Furthermore, research funders such as King Abdulaziz for Sciences and Technology and others, should increase opportunities for those carrying out stroke research to encourage them to measure and share their outcomes so others can learn from their experience, especially in rural areas. The funders also need to encourage collaboration among researchers in a variety of disciplines to ensure a feasible and appropriate evaluation for stroke care on the national level, including rehabilitation services. For patients with stroke especially in the acute stage, there is a need to improve the accessibility of health care facilities and stroke teams to ensure better outcome. The Ministry of Health and other providers should issue and widely disseminate guidelines for health care professional on stroke care, and best practice at different stages of the disease.^49^ These organizations should also encourage the use of the ICF model in all settings to ease communication and benchmarking on a national and international level regarding functioning, disability, and health.^50^

## 3. Recommendations for future research

For future research, we believe there is a need to improve the quality of conduct and reporting of national studies on stroke rehabilitation programs. Researchers from Saudi Arabia should consider using reporting guidelines such as the CONSORT, PRISMA, and STROBE to improve the quality of reporting.^51^ For example, researchers of future trials should follow the CONSORT guidelines to help in improving the quality of conducting, analyzing, and reporting results of the trials. Also, such guidelines help in minimizing bias (for example, randomization sequence generation, treatment allocation concealment, blinding, incomplete outcome data, and selective outcome reporting).

Future research in Saudi Arabia on stroke rehabilitation topics should address some of the following questions:

1. What is the current status of stroke rehabilitation services and needs?
2. What are the costs associated with stroke rehabilitation in different settings; and will using technology (such as VR) in the rehabilitation setting improve the efficiency?
3. Is there geographic variation in the quality of stroke care, including rehabilitation services?
4. Is a standardized measure for patients with stroke in different settings; for example, the ICF model, reliable and valid for use in Saudi hospitals and rehabilitation centers?
5. By using a national standardized measure for patients with stroke, do outcomes vary across the different rehabilitation settings, after adjusting for covariates?
6. For different patients with stroke (in different stages: acute, sub-acute, chronic), what is the effectiveness of rehabilitation programs that incorporate any of the following strategies: electrical stimulation, robotics, constraint-induced movement therapy, repetitive task practice, and motor imagery?
7. What role do environmental factors play on disability among patients with stroke?

In conclusion, stroke in Saudi Arabia will continue to be a major health problem associated with a higher risk of morbidity, disability, and mortality, unless the healthcare system introduces improved prevention, management, and rehabilitation services. A stroke rehabilitation program is incredibly complex, yet appears to be effective in improving patients’ quality of life and functional status. The growing evidence for the use of technology in stroke rehabilitation has been shown to be visible and safe in any setting, such as outpatient, inpatients, nursing facilities, and others. To measure the impact of these rehabilitation programs in these settings and to ensure successful implementation, measuring of outcomes is essential. The ICF-WHO model is highly recommended by international organizations to be used to provide a standardized framework in measuring functioning and disability. Finally, in this call to action paper, we have suggested some recommendations for stroke rehabilitation professionals including scientists, clinicians, and policy makers.

## Appendix

**Appendix 1:** Difference between the uses of outcome measures in stroke rehabilitation,

Name	Number of citations	Purpose	Description	Advantages	Disadvantages
Frenchay Aphasia Screening Test (FAST)^52^	240	Screening device to identify patients with communication difficulties	FAST evaluates languages in 4 noteworthy ranges: appreciation, verbal expression, perusing, and composing. Testing is engaged around a solitary, twofold sided jolt card delineating a riverside scene on one side and geometric shapes on the other and 5 composed sentences. Scores from every test territory are summed to obtain an aggregate score out of 30. Ten points are accessible for each of the cognizance and verbal expression; 5 each for perusing and composing	*Interpretability:* Age-stratified regulation of information is accessible; taking into account the appraisal of 123 people aged 20 to 81+. *Acceptability:* FAST is short and straightforward, requiring less than 10 minutes of management. *Feasibility:* FAST is easy to regulate notwithstanding during a bedside assessment. Test materials are straightforward and portable	The specificity of FAST appears, by all accounts, to be antagonistically influenced by the vicinity of visual field shortfalls, visual disregard or distractedness, lack of education, deafness, poor focus or confusion
Fugl-Meyer Assessment of Motor Recovery after Stroke (FMA)^53^	656	The FMA is intended to evaluate motor function, balance, sensation qualities, and joint function in hemiplegic post-stroke patients	The scale consists of 5 areas; motor function, sensory function, balance, joint range of motion and joint pain. Scale items are scored on the premise of capacity to complete the item utilizing a 3-point ordinal scale where 0=cannot perform, 1=performs in part, and 2=performs completely. The total possible scale score is 226 (100 for engine capacity; 24 for sensation; 14 for parity; 44 for scope of movement; and 44 for joint pain)	*Interpretability:* The interpretability of the FMA is improved by the scale’s strong establishment in all around characterized phases of motor recovery. It is broadly utilized and globally acknowledged. *Acceptability:* Administration of the entire test can be a long process; it takes 30-45 minutes. *Feasibility:* The FMA should be regulated by a prepared physical or occupational therapist. Particular equipment is not required; it is managed over an assortment of settings and can be used in longitudinal assessments	Need a prepared specialist. Takes significantly more time for evaluations
Hospital Anxiety and Depression Scale (HADS)^54^	20593	A bi-dimensional scale was developed specifically to recognize instances of depression and anxiety disorders among physically sick patients	The HADS consists of 14 items isolated into 2 subscales of 7 items each: the anxiety subscale (HADS-An) and the depression subscale (HADS-D). The respondent rates every item on a 4-point scale extending from 0 (absence) – 3 (extreme presence). Five of the 14 items were coded in reverse. The aggregate scale score is out of 42 or 21 for each of the subscales. Higher scores showed more noteworthy levels of anxiety or depression	*Interpretability:* No standards are accessible in English. *Acceptability:* The scale is convenient and simple to utilize (2-6 minutes). *Feasibility:* The HADS is easy to utilize and score	No institutionalization for age or gender has been performed. Cut-off focuses utilized are not specifically well established
Modified Ashworth Scale^55^	257	To evaluate the adequacy of hostile to spastic medication in patients experiencing different sclerosis	The unique Ashworth scale consists of 5 evaluations from 0-4. (0 = no increment in muscle tone; 1 = slight increment in muscle tone; 1* = slight increment in muscle tone; 2 = more stamped increment in muscle tone; 3 = significant increases in muscle tone; 4 = influenced part unbending in flexion or expansion)	*Interpretability:* The first Ashworth and Adjusted Ashworth scales are essential clinical measures of tone. *Acceptability:* While testing should be moderately concise, control of the affected appendage/joint may be uncomfortable for patients. *Feasibility:* No particular equipment is required	Lower levels of unwavering quality. In investigations of post-stroke patients, the most widely recognized appraisals reported are 0, 1, and 1+. The largest amounts of between spectator and intra-observer assertion are noted among patients with a 0 rating
Mini-Mental State Examination (MMSE)^56^	2771	The MMSE was created as a brief screening instrument to provide a quantitative appraisal of intellectual disability and to record subjective changes after some time	The MMSE consists of 11 basic inquiries or errands. These are assembled into 7 cognitive spaces; introduction to time, introduction to place, enrollment of 3 words, consideration and computation, review of 3 words, language, and visual development. A score of 23/24 is the most acknowledged cut-off point showing the presence of cognitive impairment	*Interpretability:* The MMSE is broadly utilized and the most acknowledged part was the cut-off scores, which were demonstrative of cognitive impairment. *Acceptability:* The test is brief, requiring approximately 10 minutes to complete. *Feasibility:* The test requires no specific equipment, requires little time and is inexpensive	It is unrealistic to distinguish adequate cut-off scores for visual or verbal memory issues Low reported levels of affectability among stroke patients
Functional Independence Measure (FIM)^57^	1293	Measures the level of understanding the disability and demonstrates the amount of help required for the person to complete the movement of activity of daily living (ADL)	Consists of 18 items: 13 motor assignments and 5 cognitive errands (considered fundamental ADL). Tasks are evaluated on a 7 point ordinal scale that ranges in total assistance (or complete dependence) to complete independence. Scores range from 18 (least) to 126 (most elevated demonstrating level of function). Scores are for the most part appraised upon admission and discharge	FIM survey the ADL FIM is used to assess impairment among adults (18-64 years); Elderly adults (65 years or older). FIM is utilized to assess mind damage, geriatrics, various sclerosis; orthopedic conditions including low back pain, spinal cord harm and stroke persistent individuals. Excellent test-retest dependability. FIM has high inward consistency and satisfactory discriminative abilities for rehabilitation patients	Standard error of estimationn mean (SEM) and insignificant recognizable. Change not built up. There is no cut-off scores. FIM is not freely accessible
Bathel Index (BI)^58^	10962	Evaluates the capacity of a person with a neuromuscular or musculoskeletal disorder to standard care	10 ADL exercises including feeding, bathing, grooming, dressing, bowel control, bladder control, toileting, chair transfer, ambulation, stair climbing. Items are appraised based on the measure of help required to complete every activity	Area of evaluation incorporates ADL; functional mobility; gait. It sets aside less time to complete the evaluation. It is an execution based measure. Equipment and preparation not required	Test-retest reliability not established
Action Research Arm Test (ARAT)^59^	275	The ARAT is a spectator appraised, execution based evaluation of upper extremity function and aptitude	The ARAT has only 19 items, which are assembled into 4 subsets: grasp (6 items), grip (4 items), pinch (6 items), and gross movement (3 items). All items are evaluated on a 4-point ordinal scale going from 0 to 3 where 0 represents no movement possible, and 3 represents normal performance of the task	*Interpretability:* As a Guttman scale, level of execution is effortlessly comprehended and contemplated. *Acceptability:* Not proper for use with proxy; negligible weight for patients. *Feasibility:* A broad gathering of items and a particular table are required. Testing must be completed in a formal setting	In patients with serious disabilities or close typical function, the scale may not be sufficiently delicate to identify changes in execution
Motor Assessment Scale (MAS)^60^	698	The MAS was created to obtain a substantial and solid method for evaluating regular motor function following stroke	The MAS consists of 8 items relating to 8 regions of motor function (recumbent to side lying, prostrate to sitting over the edge of a bed, adjusted sitting, sitting to standing, walking, upper-arm function, hand developments and propelled hand exercises). Each parameter, except for general tonus, is evaluated utilizing a 7-point pecking order of useful criteria. Score extending from 0 (most basic) to 6 (generally complex)	*Interpretability:* Scores mirror a task-oriented approach to assessment. *Acceptability:* The test is generally straightforward and brief to manage. *Feasibility:* The MAS is freely accessible in Carr et al.^66^ A time of direction and practice evaluation is prescribed preceding formal use in a clinical or exploration setting	The item “general tonus” is difficult to survey in a dependable manner. The scoring pecking order connected with the propelled hand exercises item
Six-Minute Walk Test (6MWT)^61^	48	The 6MWT is a sub maximal test of functional activity limit	The 6MWT led utilizing a lobby or tracks 100 feet long. Patients select their own power of activity and are permitted to stop and rest during the test, at their own particular pace. Performance on the 6MWT is measured by aggregate separation strolled in feet or meters within the 6 minutes.	*Interpretability:* The 6MWT is a broadly utilized apparatus that gives a quantitative measure of sub-maximal activity limit. It is concurred that age, stature, weight, and gender all freely affect the 6MWT in healthy adults. *Acceptability:* The 6MWT is moderately concise and heavily endured by patients; however, its utilization may be complicated by issues of continuance. *Feasibility:* The test is brief, modest and easy to assess	It is highly recommended that 6MWT combined with other measures for a better estimate
Stroke Impact Scale (SIS)^62^	760	Surveys health status after stroke	A 59 item measure, 8 domains are assessed: strength (4 items), hand function (5 items), ADL/IADL (10 items), mobility (9 items), communication (7 items), participation/role function (8 items). Each item is rated on a 5-point Likert scale in terms of the difficulty the patient has experienced in completing each item. Cumulative scores are obtained for every space, scores range from 0-100.	SIS assessment including ADL; cognition; communication; depression; functional mobility; gait; general health; life participation; quality of life; social relationships; social support; upper extremity function	SIS assessment is patient reported outcomes
Medical Outcomes Study Short-Form-36 (SF-36)^63^	355	The SF-36 is a non-specific health survey developed to survey health status in the all inclusive community	The SF-36 consists of 8 measurements or subscales: physical functioning, role limitations-physical bodily pain, social functioning, general mental health, role limitations-emotional, vitality, and general health perceptions. Each of the 8 summed scores is directly changed onto a scale structure 0-100 to obtain a score for every scale. Furthermore, a physical component (PCS) and mental segment score (MCS) can be obtained from the scale items	*Interpretability:* Utilization of the scale scores and synopsis part scores represents lost data and reduction in potential clinical interpretability. *Acceptability:* Fruition time is approximately 10 minutes for either the self-finished or interview managed questionnaires. *Feasibility:* It has been used as a mail survey with reasonably high completion rates reported	Higher rates of missing information have been accounted for among older patients when utilizing the self-finished type of organization. The SF-36 does not fit the era of a general synopsis score. The level of test-retest unwavering quality reported in the stroke population demonstrates that the SF-36 may not be sufficient for serial correlations of individual patients
Nottingham Health Profile (NHP)^64^	138	The NHP was intended to be a brief, subjective measure of wellbeing incorporating the social and belongings of illness	The NHP consists of 2 sections. Part I consists of 38 parameters grouped into 6 subsections: physical mobility (8 items), pain (8 items), sleep (5 items), social isolation (5 items), emotional reactions (9 items), and energy level (3 items). All parameters are weighted and given an aggregate score of 100. Part II consist of 7 items: paid livelihood, employment around the house, social life, individual connections, and sexual coexistence. These parameters are not weighted. A score out of a total of 7 is obtained by including the quantity of positive reactions. Higher scores correlate to poorer wellbeing status.	*Interpretability:* The NHP has been generally utilized in many countries. A complete client’s manual is accessible, as are populace standards. *Acceptability:* The NHP is short and basic form and requires little time to complete. *Feasibility:* The test can be managed as either a self-report or verbal postal overview	The NHP to some degree is a constrained measure. The NHP is not suited for use in the all inclusive community
Reintegration to Normal Living Index (RNLI)^65^	340	The RNLI was created as a short and straightforward approach to evaluate, quantitatively, the extent to which people who had encountered traumatic or debilitating sickness achieve reintegration	In the RNLI, 11 decisive explanations were produced. Each of these announcements is appraised by the respondent on a 10 cm visual analogue scale (VAS): does not portray my circumstance (1 or negligible reintegration) and completely depicts my circumstance (10 or most extreme reintegration). Individual item scores are summed to obtain an aggregate score out of 110, which focuses on the relative changes over time to obtain a score out of 100	*Interpretability:* There are no generally accepted standards for understanding. *Acceptability:* A short and straightforward assessment of the RNLI represents negligible patient weight. *Feasibility:* The RNLI is accessible for free. It can be utilized to evaluate longitudinal studies	The perfect composition of the subscales is questionable. Reliability and legitimacy have not been significantly studied within the stroke population
EuroQoL Quality of Life Scale (EQ5D)^66^	3144	The EQ5D is a non-specific recorded instrument, created by a multi-nation, multi-disciplinary group, used to depict well-being and esteem.	The EQ5D is a self-reported survey, consisting of 2 sections. Part I consists of 5 measurements: mobility, self-care, usual activities, pain/discomfort and anxiety/depression). Each measurement is represented by 3 articulations comparing to 3 levels: item-1 (some problems), 2 (moderate problems), and 3 (extreme problems). Part II consists of a VAS in which respondents rate their present condition of wellbeing from 0 (worst imaginable) to 100 (best possible)	*Interpretability:* EQ5D utilizes population base utility weights to obtain a standard arrangement of utility qualities for the 5-digit wellbeing state from the 5-domain index. *Acceptability:* Short and straightforward, reports of missing information are blended. *Feasibility:* It is a self-reported questionnaire that may be regulated as a postal or phone survey or in a face-to-face interview	Not suitable for use in serial evaluations of individual patients. Reliability was lower when the post-stroke intermediary respondent completed the questionnaire for understanding on the patient’s behalf
